# Individual Foraging Strategies Reveal Niche Overlap between Endangered Galapagos Pinnipeds

**DOI:** 10.1371/journal.pone.0070748

**Published:** 2013-08-15

**Authors:** Stella Villegas-Amtmann, Jana W. E. Jeglinski, Daniel P. Costa, Patrick W. Robinson, Fritz Trillmich

**Affiliations:** 1 Department of Behavioral Biology, Bielefeld University, Bielefeld, North Rhine-Westphalia, Germany; 2 Ecology and Evolutionary Biology Department, University of California Santa Cruz, Santa Cruz, California, United States of America; Institut Pluridisciplinaire Hubert Curien, France

## Abstract

Most competition studies between species are conducted from a population-level approach. Few studies have examined inter-specific competition in conjunction with intra-specific competition, with an individual-based approach. To our knowledge, none has been conducted on marine top predators. Sympatric Galapagos fur seals (*Arctocephalus galapagoensis*) and sea lions (*Zalophus wollebaeki*) share similar geographic habitats and potentially compete. We studied their foraging niche overlap at Cabo Douglas, Fernandina Island from simultaneously collected dive and movement data to examine spatial and temporal inter- and intra-specific competition. Sea lions exhibited 3 foraging strategies (shallow, intermediate and deep) indicating intra-specific competition. Fur seals exhibited one foraging strategy, diving predominantly at night, between 0–80 m depth and mostly at 19–22 h. Most sea lion dives also occurred at night (63%), between 0–40 m, within fur seals' diving depth range. 34% of sea lions night dives occurred at 19–22 h, when fur seals dived the most, but most of them occurred at dawn and dusk, when fur seals exhibited the least amount of dives. Fur seals and sea lions foraging behavior overlapped at 19 and 21 h between 0–30 m depths. Sea lions from the deep diving strategy exhibited the greatest foraging overlap with fur seals, in time (19 h), depth during overlapping time (21–24 m), and foraging range (37.7%). Fur seals foraging range was larger. Cabo Douglas northwest coastal area, region of highest diving density, is a foraging “hot spot” for both species. Fur seals and sea lions foraging niche overlap occurred, but segregation also occurred; fur seals primarily dived at night, while sea lions exhibited night and day diving. Both species exploited depths and areas exclusive to their species. Niche breadth generally increases with environmental uncertainty and decreased productivity. Potential competition between these species could be greater during warmer periods when prey availability is reduced.

## Introduction

Survival of a population is achieved through foraging success and ultimately, reproductive success. These factors will influence energy allocation to offspring and therefore, population growth. Being successful at acquiring prey is determined by prey abundance, accessibility, and species interactions, specifically competition for prey resources. Ecological niche separation can occur by organisms differing in their breeding chronology, foraging behavior, foraging time, prey type, trophic position, and life history strategies [1]–[6].

Inter-specific competition, defined as reciprocal negative effects of one species on another (either directly or indirectly mediated by changes in resource availability), is an important process determining the structure of natural communities [7]–[10]. A central tenet of Lotka–Volterra competition theory is that coexistence of two species is possible when the per capita effects of intra-specific competition on per capita rates of population growth are greater than those of inter-specific competition [11], [12]. A vast literature exists on inter- and intra-specific competition, but fewer authors have examined inter-specific competition in conjunction with intra-specific competition, e.g. [13]–[18].

Many methods have been applied to study marine top predator competition such as ecological niche models, spatial distribution, diving behavior, fatty acid analysis, stable isotopes and diet [19]–[26] but most of them with a population based approach. Considering intra-specific dynamics on a spatial and temporal scale, when studying species interactions, allows the detection of inter-specific interactions on a finer scale. To our knowledge, no such studies have been done on marine top predators.

Closely related species with similar life-history strategies often share similar niches. Non-migratory, central-place foraging species with overlapping ranges may compete for similar resources, such as prey. Among the sympatric marine mammal species with similar life-history traits and foraging habits are the otariids: fur seals and sea lions. In general, where fur seals and sea lions live in sympatry, the fur seal population is typically larger and they appear to outcompete sea lions [27].

In conditions of limited resources, competition between species implies a reduction in some population attributes, such as growth, survival or fecundity [28]. The observation that most sea lion populations are smaller when sympatric with fur seals suggests that some interspecific competition may occur. While a number of studies have examined potential competition between sympatric fur seal and sea lions, their results are mixed. Some have found ecological segregation with no trophic overlap [29]–[32], while others have found some dietary overlap [25], [33]. Most of these studies examined niche overlap in the diet of sympatric fur seals and sea lions, but few have examined overlap in terms of the spatial (both horizontally and vertically in the water column) and temporal components of foraging behavior.

On the Galapagos Islands, the Galapagos fur seal (*Arctocephalus galapagoensis*) and the Galapagos sea lion (*Zalophus wollebaeki*) coexist on several islands. Both species are endangered, and although the sea lions are more abundant (20,000–40,000 indiv.) than fur seals (10,000–15,000 indiv.) [34], the local fur seal population is usually larger where they occur sympatrically. Such is the case at Cabo Douglas, Fernandina Island, where the fur seal population is larger than that of sea lions (fur seals: 215±25 and sea lions: 42±11 individuals, [32]). While these species share similar life history strategies, sea lions breeding season is longer than that of fur seals and female fur seals wean their pups at an older age (2–3 yrs. old) than sea lions (1–2 yrs. old). Provisioning strategies also differ, fur seals foraging trip durations are usually longer and vary as a function of the lunar cycle while sea lions exhibit shorter intervals between female attendances. Therefore, time to weaning and number of feeding trips during this interval are much reduced in sea lions compared to fur seals [35].

Dellinger and Trillmich [29] studied the diet of Galapagos sea lions and fur seals in scats and vomits. They found that fur seals mostly fed on fishes of the Myctophidae and Bathylagidae families, while sea lions most important food item was sardines (*Sardinops sagax*), concluding that food-niche overlap between the two species was almost non-existent. On average, fur seals are known to dive shallower than sea lions, which holds true for Galapagos otariids [36]–[38]. Other authors have studied Galapagos sea lions and fur seals diving behavior and trophic position (stable isotopes) [23], [32], finding little to no overlap at the population level. However, sea lions have been shown to exhibit diverse foraging strategies, regarding their diving behavior and space use (shallow, intermediate and deep divers) within a population as a possible consequence of intra-specific competition [38], [39]. Acknowledging individual-level variation can benefit ecological studies as it represents a more complete description of a biological system. Individual specialization has potentially profound implications for our understanding of ecological and evolutionary processes and hence for conservation programs.

Here we examined potential foraging niche overlap in a sympatric fur seal and sea lion species at Cabo Douglas, from simultaneously collected foraging behavior data to determine the occurrence of potential competition in the spatial and temporal domain and to determine niche variability within each species. We predicted that sea lions, when in sympatry with fur seals, would exhibit similar foraging strategies to those previously found in allopatry, and that there would be an overlap between the foraging niche of shallow diving sea lions and fur seals. Alternatively, sea lions could exhibit fewer foraging strategies and the strategy most similar to fur seals would be eliminated to reduce competition.

## Methods

### Ethics statement

All research reported here, animal handling and instrumentation is in compliance with animal care regulations and applicable national laws of Ecuador, in which they were performed. This research was approved by the CARC (Chancellor's Animal Research Committee) at University of California, Santa Cruz. The appropriate animal use and care committee of Ecuador (Parque Nacional Galapagos) approved all research protocols. This work was performed under the permit No PC-11-08 and PC-043-09 of the National Park service, Galapagos.

### Field site and tagging procedures

Research was carried out during two seasons in 2009: March and October-November at Cabo Douglas, Fernandina Island (0.320° S, 91.670°W) in the Galapagos Islands. In March, 7 adult lactating female Galapagos sea lions and 6 adult lactating Galapagos fur seals were captured. In October, 10 lactating female sea lions and 11 lactating female fur seals were captured. Sea lions and fur seals were both captured with hoop nets and manually restrained for instrument attachment. Lactating females were chosen to facilitate tag recovery since they constantly return to the colony to feed their pups. Furthermore, compared to other sex/age classes, lactating females require greater energy intake from a smaller foraging range given their timely constraint to return to land and feed their offspring. Thus foraging niche competition is potentially intensified in lactating females.

To determine movement patterns at sea and diving behavior, animals were instrumented with GPS tags and time-depth recorders (TDR), either a Mk10-AF (Wildlife Computers, USA) or a Sirtrack GPS (Sirtrack, New Zealand) plus Mk9 (Wildlife Computers, USA). TDRs were programmed to sample depth, time, temperature and light level every two seconds. FastLoc GPS were set to acquire a position every 15 min. The depth resolution (accuracy) was 0.5±1% m for MK9/MK10 and mean GPS error has been estimated to 36 m [40]. Animals were also instrumented with radio transmitters (VHF) (Sirtrack, NZ) to aid in recovery on land. Instruments were mounted on mesh netting and glued to the dorsal pelage of the lower back and between the shoulders of the animals using Araldite epoxy (Araldite 2012, Huntsmann Advanced Materials, Basel, Switzerland). Sets of instruments (TDR, GPS and VHF) weighed between 0.3 and 0.7% of the study animals body mass. Animals were weighed in a sling using a tripod and a 100 kg (+/−0.2 kg precision) capacity digital scale (Kern HUS 300K 100). All study animals, except 3 sea lions and one fur seal, were recaptured after 8 to 14 days during March, and 8 to 19 days during October-November (except one fur seal: Ag5, after 51 days and one sea lion: Zw61, after 74 days). The equipment was removed by physically restraining the animals. The remaining pieces of epoxy mounts fall off within a few months during the animals' annual molt. All study animals were monitored in a subsequent field season and showed no physical impact or behavioral abnormalities as consequence of instrument deployment.

### Tracking and diving behavior analyses

To determine the animals habitat utilization and foraging range, GPS positions were decoded using the DAP processor (Wildlife Computers) and a custom software package written in Matlab (The MathWorks Inc, USA) (IKNOS toolbox) was used to filter GPS location data. The algorithm uses several criteria to remove unlikely locations: (1) realistic travel speeds of a subject between two fixes (≤6 km h^−1^ f) (2) change in azimuth between successive fixes (angle tolerance 180°), (3) on land locations and (4) time lapse between two consecutive fixes (10 min). Filtering retained approximately 80% of GPS positions, as in Jeglinski et al. [41]. Spatial analyses were performed using ArcGIS v10.0.

Dive data were analyzed in Matlab using a dive analysis program (IKNOS, Tremblay, *unpublished*), which allows for a zero-offset correction at the surface and the identification of dives based on a minimum depth and duration. During both seasons, all Mk9 and Mk10-AF recorders had a 0.5 m depth resolution and all but one recorder sampled every 2 sec (during March, a sea lion recorder: Zw33, sampled every 10 sec). The minimum depth considered to be a dive was 5 m and the minimum duration was 12 sec (10 sec for Zw33), equivalent to at least 6 depth measurements. The maximum difference considered for the length duration of tag deployments between individuals was 11 days. Only the portion of the tracking and diving behavior data for animals Ag5 and Zw61 (longest records) were used that corresponded with the same time period of the other animals.

### Statistical analyses

All statistical analyses were performed in SYSTAT 11. For all analyses, data were tested for normality using a Kolmogorov-Smirnov test and log transformed when needed. Significance level was set at P≤0.05.

#### Effect of Season on Diving Behavior

In order to increase our sample size to examine diving strategies, we analyzed data from both seasons together. We evaluated the effect of season on the diving behavior of each species, given that during the October season both fur seals and sea lions were breeding and rearing younger pups [42], [43] compared to the March season (Sea lions: n = 5 & 9; Fur seals n = 6 & 10 in March and October respectively).We performed a Principal Component Analysis (PCA) to reduce the number of variables followed by a General Linear Model (GLM) to test for the effect of season on each species. A multivariate analysis is suitable for this type of data because the diving variables are strongly correlated. Because diving variables are interdependent the use of PCA allowed us to reduce the number of original variables (17) into fewer principal components, simplifying GLM analysis.

Variables used for the PCA were mean values of the following parameters for each dive: maximum dive depth (m) and its standard deviation (SD), dive duration (sec) and SD, bottom time (sec) and SD, number of “wiggles” at the bottom of a dive (number of ascent and descent movements at the bottom of the dive, which imply foraging behavior) [44] and SD, descent, ascent rate (m s^−1^) and their SD, dive rate (dives hr^−1^), efficiency (bottom time/duration of the total dive cycle (dive duration+surface interval)) and SD. The SD of dive rate was not included as its distribution did not achieve normality after several transformations. Additionally, we used percent time diving and intra-depth zone index (% IDZ). IDZ provides an index of the tendency to repeatedly dive to a given depth [45], evidence of benthic diving. Considering 5 m was the minimum detectable depth for a dive, a user defined zone of ±10 m of the maximum depth of the previous dive was applied (i.e. 5 m above and below the previous depth) to calculate IDZ. Principal components obtained from the PCA were then used as variables in the GLM.

The PCA analysis on fur seals diving variables showed that 3 principal components (PC) explained 78.2% of the variance. The different PCs were driven by the following variables: PC1 (40.4% of the variance): dive depth, dive duration, bottom time, bottom wiggles, SD of bottom wiggles, efficiency and SD efficiency; PC2 (21.6% of variance): SD dive duration, descent rate, ascent rate and IDZ and PC3 (16.1% of variance): SD ascent rate, dive rate, % time diving and SD descent rate. The GLM performed with the above PC's as variables to test for a seasonal effect revealed that PC1 and PC3 were not significantly different between seasons and PC2 was significantly different between seasons (P = 0.03) (Table 1).

**Table 1:** Galapagos sea lions and Galapagos fur seals Principal Component (PC) loadings.

	Galapagos fur seals			Galapagos sea lions		
Diving variable	PC1	PC2	PC3	PC1	PC2	PC3
	(40.45%)	(21.6%)	(16.13%)	(65.11%)	(13.07%)	(8.38%)
Dive depth	0.813	−0.163	0.232	0.924	−0.069	0.189
SD Dive depth	0.31	0.482	0.448	0.775	−0.541	0.181
Dive duration	0.926	0.222	0.18	0.939	0.099	0.106
SD Dive duration	0.344	0.777	0.291	0.865	−0.411	−0.079
Bottom time	0.976	0.03	0.055	0.831	0.483	0.093
SD Bottom time	0.652	0.541	−0.035	0.842	0.199	−0.224
Bottom wiggles	0.934	−0.206	−0.081	0.808	0.483	0.066
SD Bottom wiggles	0.797	0.15	−0.129	0.863	0.273	−0.002
Descent rate	−0.114	−0.931	−0.216	0.894	0.235	0.152
SD Descent rate	−0.212	0.081	−0.697	0.785	−0.413	−0.248
Ascent rate	0.471	−0.732	−0.232	0.837	0.228	0.326
SD Ascent rate	−0.026	0.056	−0.73	0.823	−0.293	−0.094
Dive rate	−0.403	−0.484	0.646	−0.958	0.218	−0.135
Efficiency	0.882	−0.414	0.082	−0.656	0.633	0.251
SD Efficiency	0.784	0.072	−0.465	−0.52	−0.012	0.716
IDZ	0.649	−0.688	0.104	0.863	0.278	0.121
% time diving	−0.058	−0.325	0.757	0.176	0.524	−0.682

PC loadings from Principal Component Analysis of diving variables and their standard deviation (SD) (Mar. & Oct. 2009), Cabo Douglas, Fernandina Island. Percentages given are the percentage of variance explained by each component.

The PCA analysis on sea lions diving variables showed that 3 PCs explained 86.6% of the variance. The different PCs were driven by the following variables: PC1 (65.1% of the variance): dive depth, SD dive depth, dive duration, SD dive duration, bottom time, SD bottom time, bottom wiggles, SD bottom wiggles, descent rate, SD descent rate, ascent rate, SD ascent rate, dive rate and IDZ; PC2 (13.1% of variance): efficiency and PC3 (8.4% of variance): SD efficiency and % time diving. The GLM performed with the above PC as variables to test for a seasonal effect revealed that PC1 and PC2 were not significantly different between seasons and PC3 was significantly different between seasons (P = 0.03) (Table 1).

The variables that were affected by season in either fur seals or sea lions diving behavior were removed from further analyses. These variables were: SD dive duration, descent rate, ascent rate, IDZ, SD efficiency and % time diving. The SDs of descent and ascent rate were also removed.

#### Diving Behavior – Foraging strategies

Hierarchical cluster analyses (HCA) were conducted, one for each species separately and one with both species together, to classify diving behavior as in Villegas-Amtmann et al. [38]. The HCA was conducted using Euclidean distance and average linking method. Variables used for the sea lions HCA were the female's mean dive parameter values: maximum dive depth (m) and its SD, dive duration (sec), bottom time (sec) and SD, bottom wiggles and SD, dive rate (dives hr^−1^) and SD and efficiency (bottom time/duration of total dive cycle (dive duration+surface interval)). To further explore the existence of diverse foraging strategies, an equality of variance test was conducted between sea lions and fur seals. Greater variance would imply greater individual variability and niche width [46] and would support the existence of diverse foraging strategies. The test was performed for every diving variable mentioned above.

To compare the overall diving behavior between species, an ANOVA was performed on the means of the following diving variables: dive depth (m), dive duration (sec), bottom time (sec), number of bottom wiggles, dive rate (dives/hr) and efficiency.

#### Assessing niche overlap - night diving depth frequency distributions

To assess overlap or segregation between species, dive depth cumulative frequency histograms (5 m bin intervals) were plotted for night dives only (because fur seals predominantly dive at night [42]. Histograms were plotted for all fur seals and sea lions together and separately for each sea lion diving strategy found in the HCA. Based on the cumulative percentage, the percentage of night dive depths that overlapped between fur seals and each sea lion diving strategy was determined. To evaluate differences in night dive depth frequency distributions between species, two chi-squared tests were performed comparing frequencies between 0–40 m (where most of the overlap occurs) and 50–110 m (where the rest of the overlap occurs). Additionally, the percentage of dives that occurred at night was calculated for all fur seals, sea lions and each sea lion diving strategy.

#### Assessing niche overlap - time of night dives

To further examine the potential competition between species, the frequency and percentage of dives that were occurring at the different night hours were calculated. The mean dive depth of the dives that occurred at the most frequented night hours was also calculated (only dives between 0–130 m -where species overlap occurred- were considered). Furthermore, we calculated the percentage of night dives that occurred at the different night hours between 0–30 m for fur seals vs. sea lion shallow diving strategy, and 0–40 m for fur seals vs. sea lion intermediate and deep diving strategies (where the greatest overlap occurred between fur seals and the different sea lion strategies). Finally, the percentage of dives (from all dives, day and night) that occurred at the overlapping night hours for fur seals and every sea lion diving strategy group were calculated.

#### Dive depth maximum efficiency

Dive depth maximum efficiency was calculated by splitting the diving depths of each species into 10 m bins (depth range). Mean dive depth maximum efficiency (bottom time/duration of the total dive cycle (dive duration+surface interval)) was then calculated for those depths and plotted against depth range. Dive depth maximum efficiency analysis was performed on all dives (day and night) for all animals. Prior to the dive depth efficiency calculation, surface intervals were filtered to eliminate values that included the interval after a foraging bout and the haul-out period (extremely long surface intervals). Surface interval histograms were plotted using cumulative percentages. All surface intervals after the cumulative percentage line had reached an asymptote were eliminated (all surface intervals ≥2.85 min for fur seals and ≥22 min for sea lions). Elimination of surface intervals was further corroborated by the existence of a positive linear trend between dive duration and surface interval.

#### Spatial analyses

To investigate the spatial segregation or overlap of foraging activity between the two species, we identified the position of each dive based on a linear interpolation of the processed tracking data and utilized geo-referenced foraging locations, following Jeglinski et al. [41]. In order to do that, trips to sea (here defined as exceeding 45 min wet time) were determined based on wet/dry sensor data of TDRs using a custom written MatLab function. GPS data were split in separate trips, assigning the closest on land GPS position in time to the start and end of each trip. GPS tracks were interpolated using a hermite spline (Tremblay et al. 2006; Kuhn et al. 2010). A land avoidance algorithm was applied to interpolated tracks to adjust positions that were on land to nearby water positions. Each dive was associated with a GPS location using a time based linear interpolation between track points. For subsequent analyses data were converted into the Universal Transverse Mercator (UTM) coordinate system. A kernel density analysis using a 5 km bandwidth (ArcGIS v10.0) was run using the dive locations of each species to identify regions of concentrated dive effort (presumed foraging activity). The 95% volume contour was then calculated to estimate the foraging range of each species. To identify the potential region of overlap between the two species, the intersection (overlap) between the two species was calculated. This procedure was also repeated for each sea lion diving strategy separately. Foraging range was calculated for all dives (day and night) and for night dives separately.

## Results

### 

#### Diving behavior – foraging strategies

**Figure 1:**
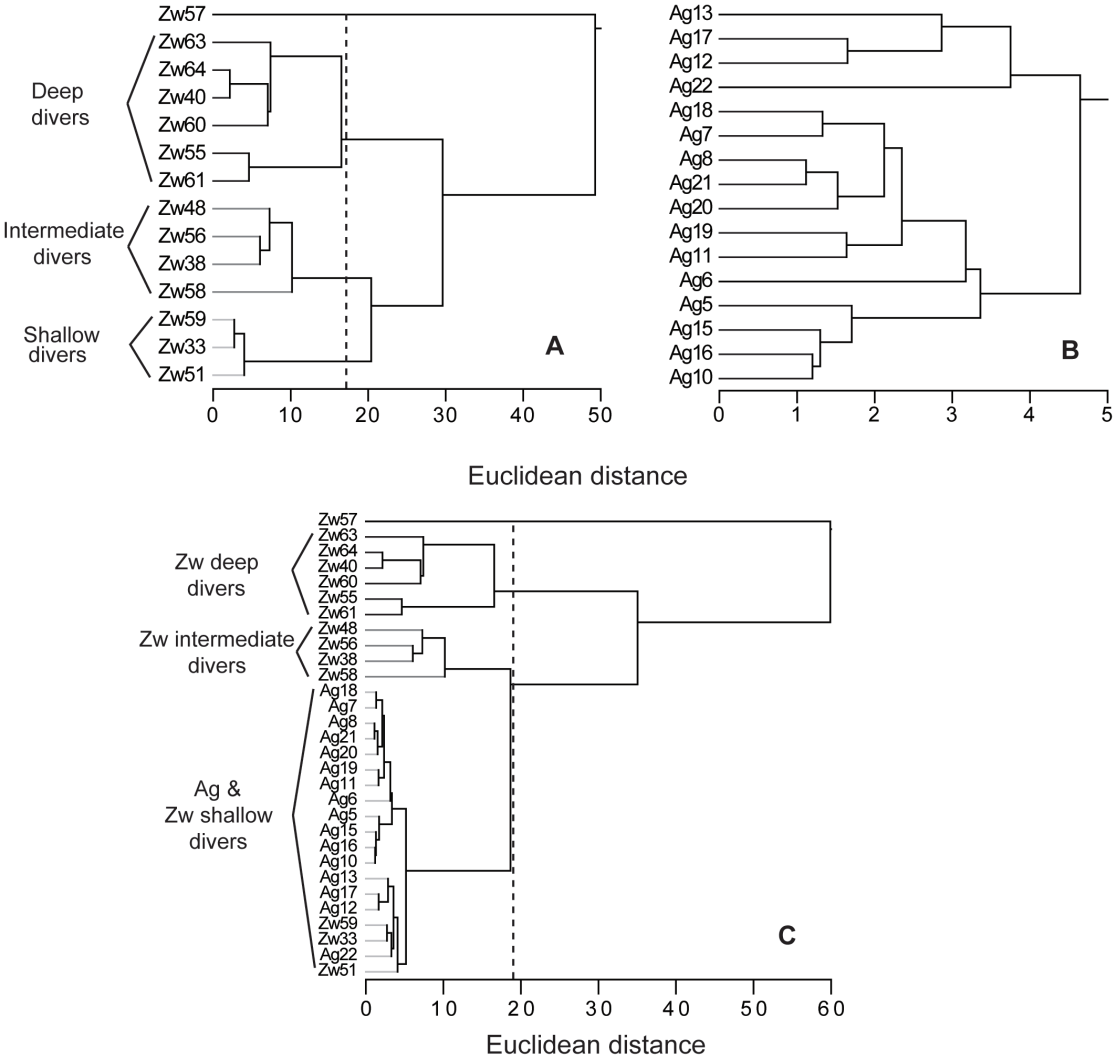
Galapagos sea lions and Galapagos fur seals diving variables cluster trees. Galapagos sea lions (Zw) and Galapagos fur seals (Ag) cluster trees of diving variables from Hierarchical Cluster Analysis (Mar. & Oct. 2009) at Cabo Douglas, Fernandina Island. A. Sea lions, B. Fur seals and C. Sea lions and fur seals together. Line indicates the Euclidean distance chosen to define groups.

The HCA of sea lion diving variables produced 4 groups which, essentially differed in depth use. Sea lion groups were classified as: Shallow, intermediate and deep divers. A fourth group with only one animal (Zw57) that dived exceptionally deep was considered an outlier and therefore removed from further analysis. Shallow divers (Zw33, Zw51 & Zw59) exhibited the shallowest dive depth (mean: 19.7±3.6 m), shortest dive duration (1.8±0.2 min), greatest dive rate (18.7±0.6 dives/h) and greatest dive efficiency (0.39). Intermediate divers (Zw38, Zw48, Zw56 & Zw58) presented intermediate dive depth (53.9±13.9 m), dive duration (2.5±0.4 min) and dive rate (13.2±0.5 dives/h). Deep divers (Zw40, Zw55, Zw60, Zw61, Zw63 & Zw64) showed the deepest dive depth (103.0±18.3 m), longest dive duration (3.6±0.8 min) and lowest dive rate (8.7±1.1 dives/h). The Euclidean distance considered for the group classification was 17–19 based on the cluster tree produced from the analysis and the diving variables similarities (Table 2 & Fig. 1).

**Table 2:** Galapagos sea lions and Galapagos fur seals diving variables.

ID	Mean dive depth (m)	Mean dive duration (min)	Mean bottom time (min)	Mean # bottom wiggles	Mean descent rate (m/s)	Mean ascent rate (m/s)	Mean dive rate dives/hr	IDZ %	Max. dive depth (m)	Max. dive dur. (min)	Max. bottom time (min)	Mass (kg)	% time diving when at sea	Total # of dives	Efficiency
**Galapagos sea lions**															
Zw33	18.5±18.3	2.1±0.9	1±0.7	2.9±2	0.6±0.3	0.6±0.3	19±8.7	23.6	256.5	8.5	4.2	63.4	43.8	1622	0.4±0.2
Zw38	57.8±63.6	2.8±1.8	1.2±1	8.9±9.1	1.2±0.5	0.9±0.4	13.2±7.5	25.6	423.0	9.7	4.6	73.8	63.0	2672	0.3±0.2
Zw40	108±106.2	3.9±2.8	1.6±1.4	8.4±8	1.1±0.6	1.1±0.7	8.5±5.3	39.9	423.0	10.7	5.6	66.2	56.8	2540	0.3±0.1
Zw48	70±85.9	2.9±2.2	1±0.8	5.7±5.2	0.9±0.6	0.8±0.5	12.6±10.5	27.2	343.5	9.2	5.1	62.8	60.7	1650	0.3±0.2
Zw51	16.89±10.4	1.7±0.8	1.1±0.7	6.1±4.4	0.8±0.3	0.9±0.3	17.9±9.7	28.4	148.0	6.8	4.0	68.6	58.9	2893	0.4±0.2
Zw55	89.43±138.4	2.9±2.4	1±0.8	6.2±7.1	1.1±0.6	1±0.5	9.1±7	27.8	584.0	11.4	6.2	89.8	39.2	1828	0.3±0.2
Zw56	50.9±80.3	2.5±1.8	1.2±0.7	6.8±4.1	0.9±0.5	0.9±0.4	13.5±10.4	30.5	394.0	8.8	4.2	61.6	52.5	2725	0.4±0.2
Zw57	203.2±151	5.2±2.7	1.7±1	8.4±7.6	1.6±0.5	1.5±0.5	6.2±4	52.2	517.5	10.4	4.7	61.2	50.8	1415	0.2±0.2
Zw58	36.8±56.1	2±1.4	0.8±0.6	4.3±3.6	0.8±0.4	0.8±0.4	13.8±11.1	20.4	414.0	9.9	4.0	64.6	36.9	1512	0.3±0.2
Zw59	23.7±24.8	1.7±1	0.9±0.5	4.3±2.4	0.9±0.3	1±0.3	19.1±10.2	18.5	173.0	5.5	3.0	72.7	56.1	2553	0.4±0.2
Zw60	103.1±84.8	3.6±1.9	1.6±0.9	12.3±8.8	1.4±0.6	1.4±0.7	9.1±5.4	55.6	430.0	9.1	3.9	51.4	54.0	1485	0.3±0.2
Zw61	106±160	3±2.8	0.9±0.8	5.4±4.1	1±0.7	1.1±2.4	9±8.4	30.0	593.0	11.2	4.6	74.6	28.4	6022	0.2±0.2
Zw63	127.4±91	4.7±2.2	2.1±1.2	14.1±10.4	1.4±0.5	1.4±0.5	6.9±4.4	44.9	382.0	11.5	6.4	57.4	51.6	1438	0.3±0.2
Zw64	114.1±103.8	4±2.3	1.5±1	9.4±7.2	1.2±0.7	1.1±0.6	8±4.4	46.8	343.5	10.0	5.5	79	55.4	1546	0.2±0.1
AVG	**78.3**	**3.0**	**1.2**	**7.4**	**1.1**	**1.0**	**11.9**	**33.4**	**387.5**	**9.4**	**4.7**	**67.6**	**52.1**	**2094.6**	**0.3**
SD	51.3	1.1	0.4	3.1	0.3	0.3	4.4	12.1	132.2	1.7	0.9	9.7	7.6	679.2	0.1
CV(%)	65.5	36.0	30.7	42.4	26.2	25.3	36.6	36.2	34.1	17.8	19.9	14.4	14.7	32.4	19.9
**Galapagos fur seals**															
AG5	32.4±24	1.8±1.1	0.9±0.6	5.4±4	1±0.4	1±0.4	10.4±10.2	49.5	131.0	5.5	4.0	25.4	19.8	4968	0.3±0.2
AG6	32.9±26.6	2±1	0.8±0.6	3.9±2.8	0.8±0.3	0.8±0.4	6.9±6.4	34.7	105.0	4.5	2.9	23.4	13.4	657	0.2±0.2
AG7	28.8±16.9	1.9±1	1±0.8	6.4±5.4	1±0.4	1.1±0.4	8.2±7.1	38.6	79.0	4.7	4.3	27.8	14.5	828	0.3±0.2
AG8	30±14.9	1.6±0.8	0.8±0.6	5.9±3.9	1.2±0.4	1.1±0.4	10.3±11.1	40.5	98.0	5.7	3.7	27.2	17.8	682	0.3±0.2
AG10	36.6±20.6	2±1	1±0.7	5.6±4.2	1±0.4	1.1±0.4	13.1±10.1	43.7	106.5	5.5	3.7	30.2	28.5	1186	0.3±0.2
AG11	33.8±22.5	2.2±1.3	1.1±0.8	5.9±4.7	0.8±0.4	1±0.3	8.3±6.7	31.9	87.0	5.8	3.6	28.6	18.2	1055	0.3±0.2
AG12	28.3±17.4	1.5±0.9	0.7±0.6	4.7±3.4	1.2±0.4	1.1±0.3	15.8±11.9	43.3	97.0	5.0	3.2	25.0	32.0	1641	0.3±0.2
AG13	23.9±19.1	1.3±1	0.5±0.5	2.9±2.7	1±0.3	0.8±0.3	20.6±19.8	28.5	118.0	3.8	2.5	34.0	41.5	806	0.2±1
AG15	40.2±22.1	1.8±0.9	0.8±0.6	4.9±3.4	1.2±0.4	1.3±0.4	14.4±9.7	49.6	97.0	4.5	2.7	31.0	28.3	1469	0.3±0.2
AG16	39.1±19.4	2.3±1	1.3±0.7	6.1±3.7	1.1±0.4	1.2±0.4	13.2±7.6	56.6	88.5	4.6	3.1	25.6	37.2	1430	0.4±0.2
AG17	25.7±14.9	1.3±0.7	0.6±0.5	4.3±3.1	1.2±0.4	1.1±0.4	17.8±15	44.6	89.0	4.4	2.3	22.0	26.6	1498	0.3±0.2
AG18	28.3±18.7	1.5±0.8	0.7±0.6	4.1±3.5	1.1±0.5	1±0.4	6.2±6.3	36.1	88.0	3.8	3.1	34.0	9.6	230	0.2±0.2
AG19	35±17.8	1.9±0.7	1±0.6	6.3±3.7	1.2±0.4	1.2±0.4	8.2±8.3	57.7	81.0	3.8	2.8	23.2	15.3	706	0.3±0.2
AG20	31.7±19.7	1.5±0.9	0.7±0.6	4.7±3.4	1.1±0.4	1.2±0.4	10.7±9.9	39.8	96.5	5.1	3.2	28.2	18.0	782	0.3±0.2
AG21	28.8±17.3	1.5±0.9	0.7±0.6	4.4±3.2	1.1±0.4	1.2±0.4	11.9±11.3	40.0	76.0	5.4	4.2	29.4	20.1	760	0.3±0.2
AG22	20.7±16.9	1.2±1	0.5±0.7	2.6±2.5	1±0.5	0.9±0.4	10.8±9.2	20.0	78.5	5.3	3.9	30.4	20.7	529	0.2±0.2
AVG	**31.4**	**1.7**	**0.8**	**4.9**	**1.1**	**1.1**	**11.8**	**41.0**	**94.7**	**4.8**	**3.3**	**27.8**	**23.7**	**1048.7**	**0.3**
SD	5.7	0.3	0.2	1.2	0.1	0.1	4.0	9.9	14.9	0.7	0.6	3.6	9.6	557.7	0.1
CV(%)	18.0	19.2	27.3	24.1	12.2	13.7	33.9	24.2	15.8	13.8	18.8	13.0	40.5	53.2	20.9

Mar. & Oct. 2009, Cabo Douglas, Fernandina Island (values are expressed as mean +/− standard deviation).

**Figure 2:**
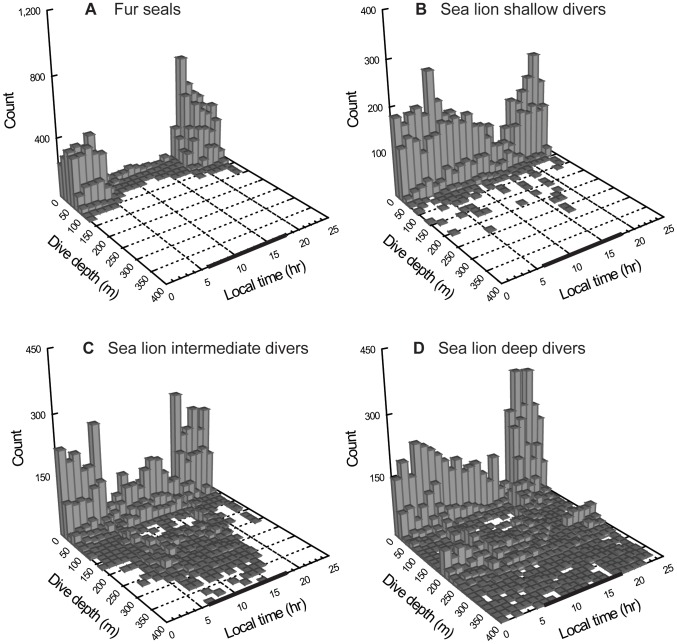
Galapagos fur seals and Galapagos sea lions frequency of mean dive depths. Galapagos fur seals (A) and Galapagos sea lions diving strategies (B–D) frequency of mean dive depths during day and night (Mar. & Oct. 2009) at Cabo Douglas, Fernandina Island. Black bar shows day hours.

Sea lions from all groups exhibited day and night diving with 63.1% of dives occurring at night. Shallow divers exhibited 55.6%, intermediate divers 54.9% and deep divers 80.3% of their dives during the night (Fig. 2).

The HCA of fur seals diving variables showed that all animals clustered together at a Euclidean distance of 4.8. Comparing this to the grouping Euclidean distance of sea lions, fur seals exhibited only one diving strategy (Table 2 & Fig. 1). As expected, almost all of the fur seal dives were performed during the night (95.6%; Fig. 2).

The HCA of the diving variables of both species together produced four groups: At the same Euclidean distance as considered for sea lions HCA (17–19), all fur seals and sea lion Shallow divers clustered together, whereas sea lion Intermediate divers, sea lion Deep divers and Zw57 (sea lion outlier) formed separate groups. The existence of diverse foraging strategies in sea lions compared to fur seals was further supported by the “equality of variance test” results (performed on all animals). The variance of all diving variables were greater for sea lions than fur seals and all variables except dive rate and efficiency were significantly different between species (P<0.001 for dive depth, SD dive depth, dive duration and SD bottom wiggles; P = 0.05 for bottom time; P = 0.003 for SD bottom time and P = 0.001 for bottom wiggles) (Table 2). Additionally, the coefficient of variance (CV) of the three sea lion foraging strategies was significantly smaller (shallow: 18.1%, intermediate: 25.8% and deep 17.7%) than the CV of all sea lions pooled together (65.5%), further supporting the existence of diverse foraging strategies in sea lions.

The ANOVA performed on the diving variables between species showed that dive depth (F-ratio = 13.22, N = 30, P = 0.001), dive duration (F-ratio = 20.76, N = 30, P<0.0001), bottom time (F-ratio = 13.97, N = 30, P = 0.001) and number of bottom wiggles (F-ratio = 8.65, N = 30, P = 0.006) were significantly greater in sea lions than fur seals. Dive rate and efficiency were not significantly different between species.

### Assessing niche overlap

#### Night diving depth frequency distributions

84.7% of sea lion night dives overlapped with fur seals diving depth range, shown in their dive depth frequency histograms. Given that there was greater variability and 3 foraging strategies (shallow, intermediate & deep divers) in the sea lions diving behavior compared to fur seals, competition between these species was evaluated considering all the fur seals and each sea lion foraging strategy separately. Sea lion shallow divers nocturnal depth range (55.6% of total number of dives) overlapped completely with fur seals diving depth range: 0–120 m, whereas 96.3% of sea lion intermediate divers (52.9% of total dives) and 72.4% of sea lion deep divers (58.1% of total dives) nocturnal depth range overlapped with that of fur seals.

**Figure 3:**
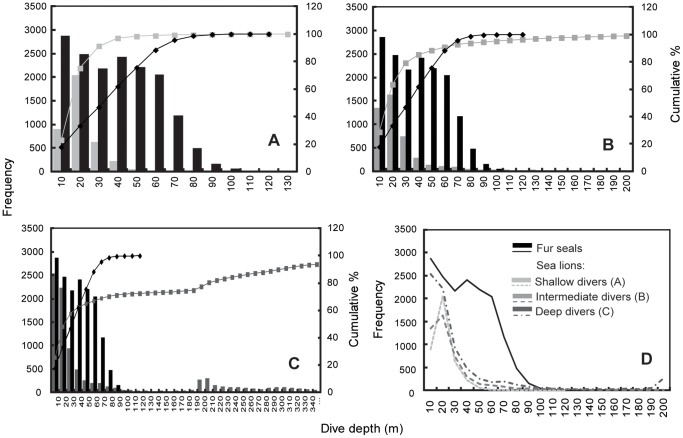
Galapagos sea lions and Galapagos fur seals dive depth histograms. Galapagos sea lions and Galapagos fur seals dive depth histograms (Mar. & Oct. 2009) at Cabo Douglas, Fernandina Island. The different sea lion diving strategies are shown separately (A, B & C, grey columns) in comparison to fur seals (black columns in A–C). (D) The 3 strategies together with fur seals.

All three groups of sea lions night-time dive depth frequency histograms exhibited a peak between 0–30 m, beyond which little diving occurred. The fur seals dive depth frequency histogram exhibited two peaks, a larger one between 0–30 m (46.8% of their dives occur in this range) and the second one in the range of 40–80 m (where 51.8% of their dives occur). Histograms show that most diving depth overlap between fur seals and sea lions occurred in the range of 0–40 m (Fig. 3). Consequently, in the first 40 m of the water column, where most of the dive depths overlap, 61.8% of fur seal dives occurred, 97% of sea lion shallow divers dives, 85.3% of sea lion intermediate divers dives and 61.8% of sea lion deep divers dives.

Chi-squared tests on dive depth distributions showed that for both depth zones (0–40 m and 50–110 m), distributions were significantly different between fur seals and each sea lion diving strategy (P<0.0001 for all tests).

#### Time of night dives

**Figure 4:**
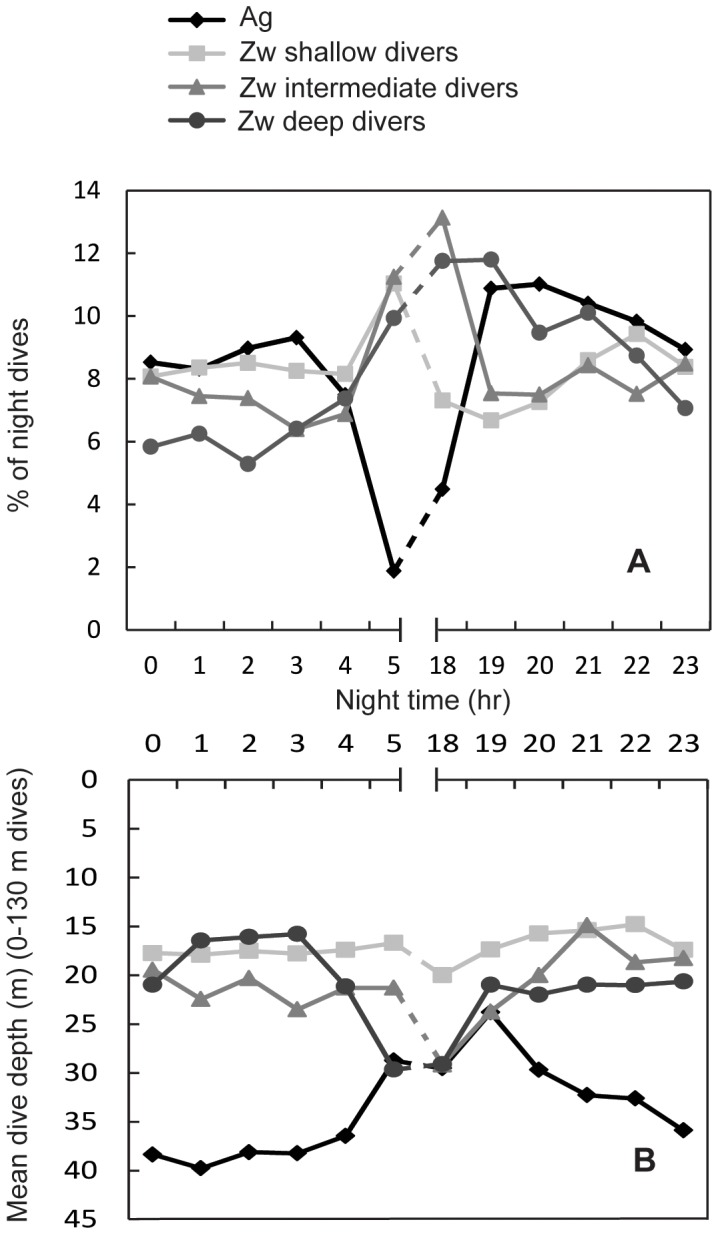
Galapagos sea lion and Galapagos fur seal percentage and mean depth of overlapping night dives. Percentage of dives (A) and mean dive depth of dives (B) for three Galapagos sea lion diving strategies and Galapagos fur seals covering the range of fur seals dive depth (0–130 m) during night time (Mar. & Oct. 2009) at Cabo Douglas, Fernandina Island.

Sea lions exhibited the greatest proportion of their dives just after 18:00 (after sunset) (intermediate and deep divers) and just before 05:00 (before sunrise) while fur seals were least active during those hours. Mean dive depths (0–130 m where overlap occurs) of sea lions were closest to fur seals mean dive depths at 19:00 (sea lion intermediate and deep divers) and at 5:00 (sea lion deep divers) (Figs. 2 and 4).

The greatest overlap in the time of day when most dives occurred between fur seals (19% of total dives) and sea lion shallow divers (10% of total dives) occurred at 22:00 and 21:00 (Table 3). Most sea lion shallow strategy dives (96%) at these hours occurred between 0–30 m depths where 64% of fur seal dives occurred. However, it is important to consider that sea lion shallow divers exhibited the greatest percentage of night dives at 5:00 (6% of total dives) when fur seal diving activity is very limited (2% of total dives).

**Table 3:** Galapagos sea lions and Galapagos fur seals % of night dives by night hour.

	Fur seals	Sea lion Shallow divers	Fur seals	Sea lion Intermediate divers	Sea lion Deep divers
	% night dives 0–30 m		% night dives 0–40 m		
18	59.4	83.4	69	**75.4**	**62.7**
19	**69.9**	93.9	**81.5**	86	**72.7**
20	**53.4**	95.1	**71**	93.3	64.7
21	**46.9**	**96.1**	**63.2**	**98.9**	**67.4**
22	**51.4**	**96.5**	**65.6**	94.8	65.3
23	40.3	91.5	58.7	**92.7**	61.9
24	36.8	93.7	54.1	88.6	52.9
1	35.2	90.1	49.4	84.3	62.5
2	37.8	**89.2**	53	86.3	52.2
3	35.2	86.4	52.8	84.2	66.2
4	41.1	89.6	53.6	83.7	63.3
5	57.2	**89.8**	67.3	**75.6**	**50**

Dives between 0–30 m and 0–40 m, where overlap occurs. Mar. & Oct. 2009 at Cabo Douglas, Fernandina Island. In bold are the hours where the greatest % of night dives occur.

Overlap in the timing of dives between fur seals (20% of total dives) and sea lion intermediate divers (9% of total dives) occurred at 21:00 and 19:00 (Table 3). These dives occurred within 0–40 m, the depth range of 93% of sea lion intermediate divers and 73% of fur seal night dives. Similarly to sea lion shallow strategy, most night dives for this sea lion group (which are 55% of total dives) occurred at 18:00 and 5:00 (12% of total dives), the two night hours with the least diving activity in fur seals (6% of total dives).

Overlap between fur seals (31% of total dives) and sea lion deep divers (19% of total dives) occurred at 19:00, 20:00 and 21:00 (Table 3). These dives also occurred within 0–40 m depth, the depth range of 69% of sea lion deep divers and 72% of fur seal night dives. In contrast to the other sea lion groups, 19:00 is when most dives occurred for both species (7% of total sea lion deep strategy dives and 10% of fur seal total dives) and when their mean dive depths are closer (Fig. 4). Sea lion deep divers also exhibited a large percentage of dives at 18:00 and 5:00 (13% of total dives) in contrast with fur seals.

The percentage of dives from all dives (day and night) that occur at these overlapping depths and times between species, are, for fur seals at 19:00, 10.4%; 20:00, 10.5%; 21:00, 9.9% and 22:00, 9.4%. The percentage of dives from all dives for sea lion shallow divers was, at 21:00, 4.7% and 22:00, 5.2%; for sea lion intermediate divers, at 21:00, 4.4% and for sea lion deep divers, at 19:00, 7.3%, 20:00, 5.9% and 21:00, 6.3%.

### Dive depth maximum efficiency

**Figure 5:**
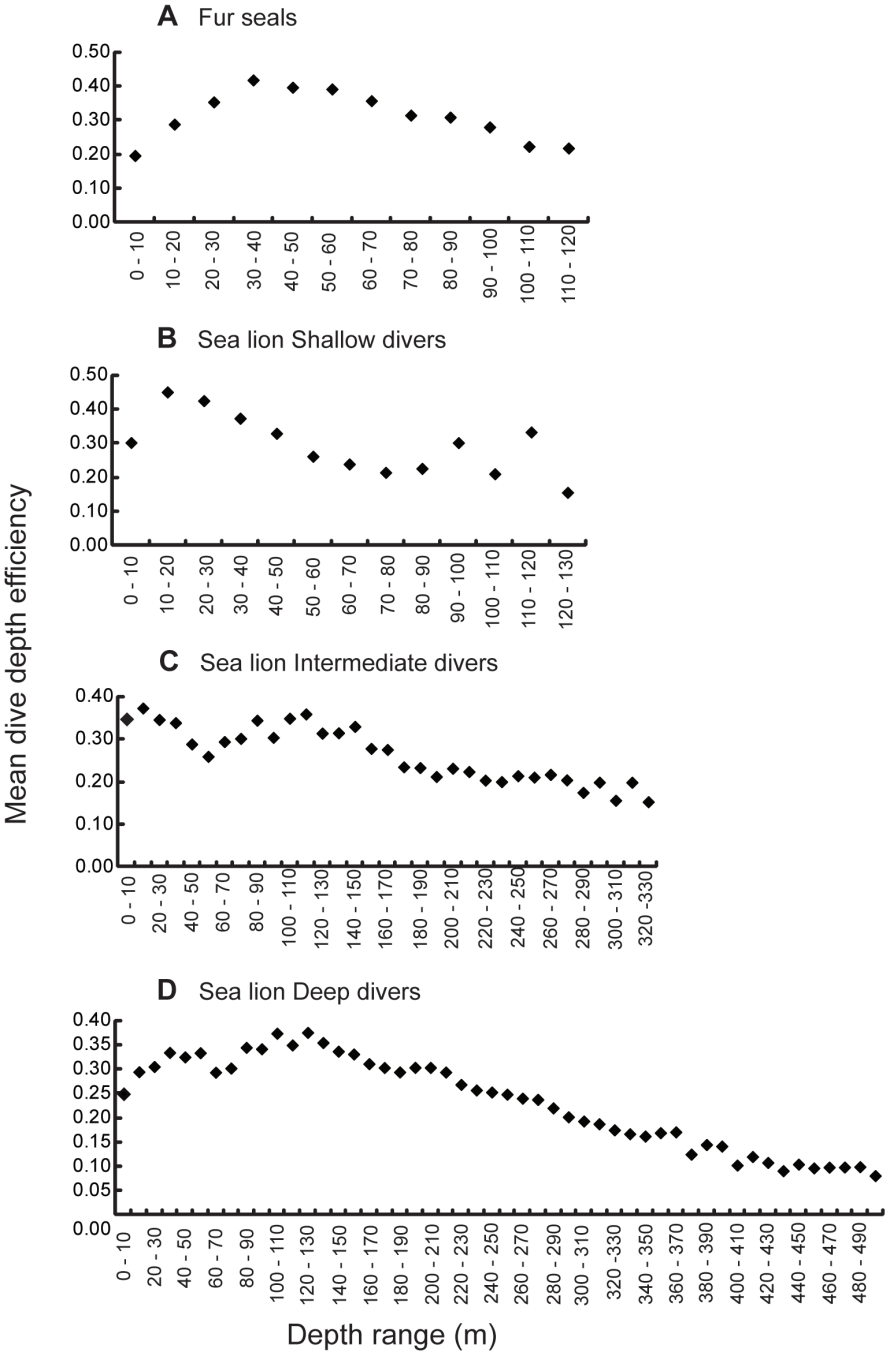
Galapagos sea lions and Galapagos fur seals dive depth maximum efficiencies. Galapagos sea lions diving strategies (B–D) and Galapagos fur seals (A) mean dive depth maximum efficiencies (Mar. & Oct. 2009) at Cabo Douglas, Fernandina Island.

Compared to sea lions, fur seals exhibited a narrower range of dive depths where the diving efficiency was maximized. Fur seals exhibited one dive depth peak of maximum efficiency within the range of 30–40 m. Sea lions exhibited two peaks, one between 10–20 m and the second one at 80–120 m. When looking at sea lion diving strategies separately, shallow divers presented two peaks of maximum efficiency at 10–20 m and 90–120 m. Intermediate divers presented two peaks: at 10–20 m and 110–120 m and deep divers one peak at 100–130 m (Fig. 5).

### Spatial analyses

**Figure 6:**
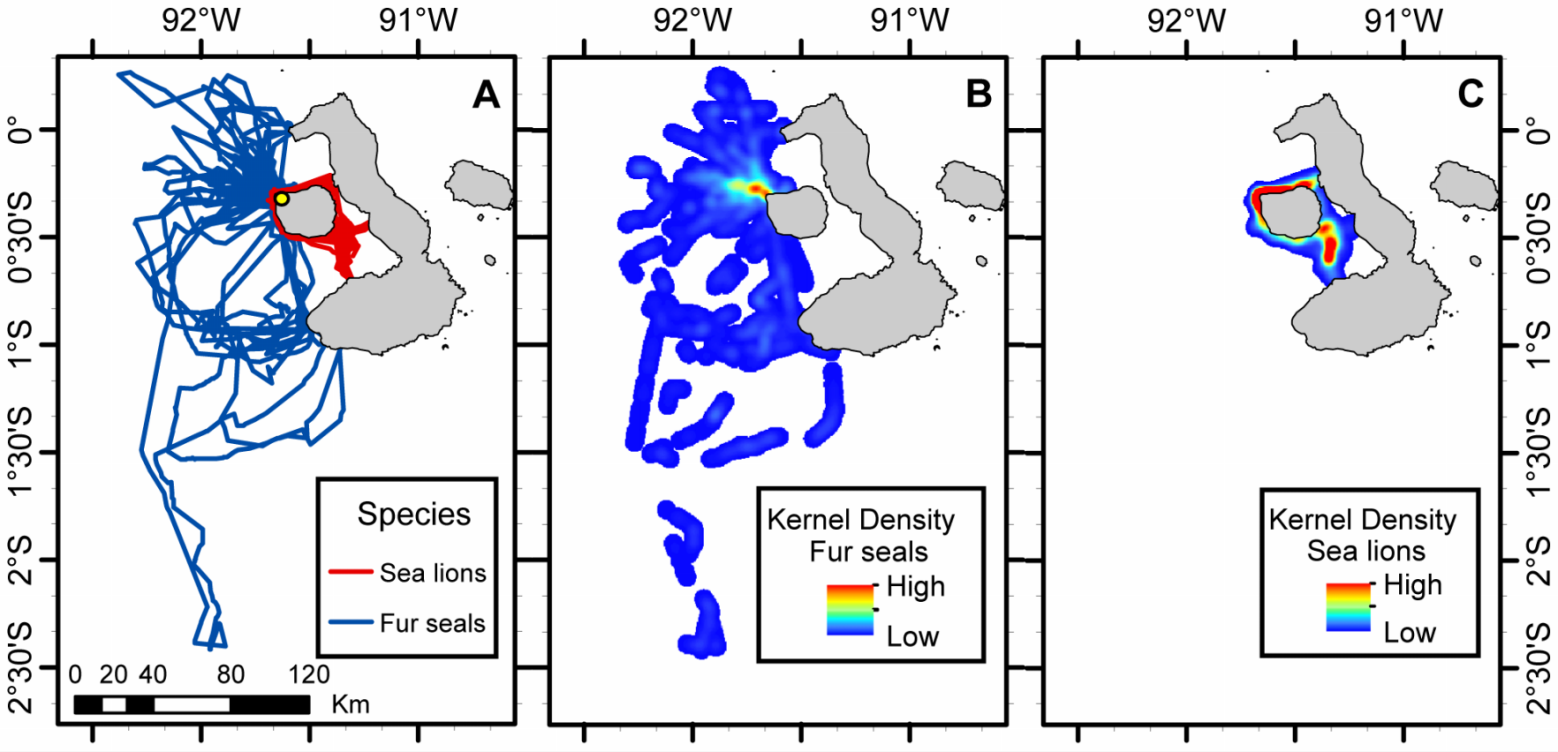
Galapagos sea lions and Galapagos fur seals foraging trips and foraging ranges. Galapagos sea lions and Galapagos fur seals foraging trips (A) and foraging ranges based on 95% contour kernel analysis (B & C) (Mar. & Oct. 2009). The study colony Cabo Douglas, Fernandina Island is indicated by a yellow circle.

**Figure 7:**
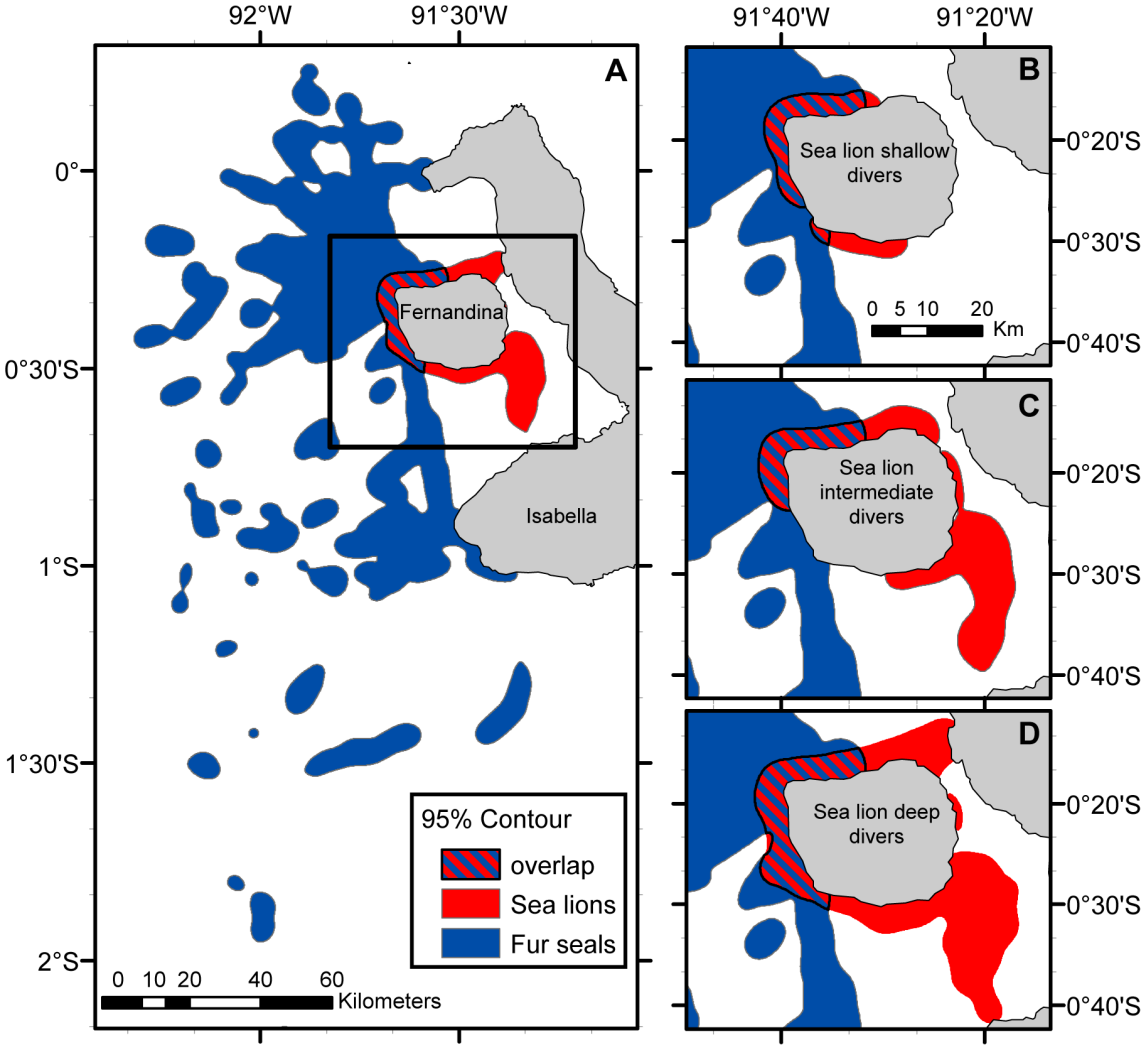
Galapagos sea lions and Galapagos fur seals foraging range overlap. Galapagos sea lions and Galapagos fur seals overall foraging range overlap (A) and overlap between fur seals and each sea lion diving strategy (B–D) based on 95% contour kernel analysis (Mar. & Oct. 2009) at Cabo Douglas, Fernandina Island.

The foraging range (95% volume contour kernel analysis) of fur seals was 5999 km^2^, 7 times greater than that of sea lions, 840 km^2^ (Table 4 & Fig. 6). Among the different sea lion groups, the foraging range was greater for sea lion deep divers (951 km^2^), followed by intermediate divers (645 km^2^) and shallow divers (320 km^2^) (Table 4). Fur seals foraged coastally and offshore to the north, northwest, west and southwest of Fernandina Island with the greatest concentration of diving locations to the northwest of the island. Sea lion deep and intermediate divers foraged coastally and offshore to the north, south and southeast of Fernandina Island, while sea lion shallow divers only foraged coastally to the north, west and south of Fernandina Island. Sea lions also exhibited the greatest density of dives north of the island. The percentage of foraging range overlap between fur seals and all sea lions as well as fur seals and each sea lion diving strategy was almost identical when considering all dives (day and night) or night dives alone. A greater percentage of sea lion's foraging range overlapped with that of fur seals, for all 3 sea lion groups (20.70–37.67%), than fur seals foraging range (2.93–5.33%) with each of sea lions diving groups. This overlap was greater between fur seals and sea lion deep divers, 5.33% of fur seals night foraging range overlapped with sea lion deep divers range and 37.67% of sea lion deep divers foraging range overlapped with that of fur seals (Table 4 & Fig. 7).

**Table 4:** Galapagos sea lions (Zw) and Galapagos fur seals (Ag) foraging range and % foraging range overlap.

		Day & night overlap		Night overlap	
	Foraging range (Km^2^) (Day & night)	% of Zw range	% of Ag range	% of Zw range	% of Ag range
Ag x Zw	292.30	34.82	4.87	34.92	4.94
Ag x Zw shallow d. overlap	217.85	25.95	3.63	26.49	3.75
Ag x Zw interm. d. overlap	194.02	23.11	3.23	20.70	2.93
Ag x Zw deep d. overlap	314.13	37.42	5.24	37.67	5.33
Ag	5998.68				
Zw	839.57				
Zw shallow divers	319.52				
Zw intermediate divers	645.39				
Zw deep divers	950.97				

Foraging range is based on kernel density estimates of dive locations (Mar. & Oct. 2009) at Cabo Douglas, Fernandina Island.

## Discussion

### Diving behavior – foraging strategies

Sea lions exhibited greater individual variability in their diving behavior compared to fur seals. Within the archipelago, more central to their distribution, adult female sea lions exhibited 3 foraging strategies, suggesting intra-specific competition [38], [39]. In this study where sea lions are at their western distribution they also exhibited 3 distinct foraging strategies. In contrast, fur seals exhibited a rather uniform diving behavior with only one foraging strategy. This suggests greater intra-specific competition may be a common feature in the foraging behavior of sea lions. In contrast, the single foraging pattern of fur seals suggests that prey may be quite abundant in the deep scattering layer of this area. Intra-specific competition could be related to sex or age. However, because our study included only adult females, we were unable to test these parameters.

Sea lions dove deeper, longer, spent more time at the bottom of their dives and greater number of bottom wiggles than fur seals, as expected from previous work [36], [43]. Sea lions are significantly larger than fur seals and larger animals have proportionately greater oxygen stores and therefore, greater breath-hold capacity [47]–[49].

The west coast of the archipelago, where this study was carried out, is the most productive area of the Galapagos archipelago [50], [51]. The fur seal and sea lion rookeries are located within the upwelling region of the cold Cromwell countercurrent. Although in a productive area, sea lions exhibited greater foraging effort as shown by the greater percentage of time spent diving compared to fur seals, possibly due to reduced prey availability or different prey distribution. This suggests that food resources might be limited or less accessible for sea lions here in the west as well as for their central distribution in the Galapagos archipelago [38], [39]. Sea lions in the western part of the archipelago are known to feed on deep water pelagic and demersal fish such as sardines (*Sardinops sagax*), jack mackerel (*Trachurus symmetricus*) and *Chlorophtalmus* sp. [23], [29]. In contrast, fur seals feed on shallower waters, on prey from the deep scattering layer when they migrate to the surface during night, e.g. myctophids, bathylagids and cephalopods [23], [29], [52]. Furthermore, sea lions are known to forage over the shelf [32], [38]. In the western region of the Galapagos archipelago the shelf habitat is very limited, the only regions where benthic habitat is accessible to sea lions are close to the coast of Fernandina and in the Bolivar channel between Fernandina and Isabela islands.

Costa & Gales [53] postulated that increased foraging effort may explain why many pinnipeds and penguins that feed benthically have small stable or declining populations, while the many epipelagic divers have large stable and or increasing populations. This appears to hold true at Fernandina Island, where the fur seal population is significantly larger than the sea lion population.

### Assessing niche overlap

#### Night diving depth frequency distributions

Fur seals and sea lion shallow divers exhibited similar diving behavior as they clustered together in the HCA, 100% of their night dives overlapped and 50% of the sea lion shallow strategy dives occurred at night; therefore, they could potentially compete. In addition, because 80% of the sea lion deep strategy dives occurred at night, and they exhibit shallow and deep dives, this sea lion group foraging niche could also potentially overlap with fur seals. Sea lion deep divers might exhibit shallow and deep dives to potentially avoid competition with other sea lion strategies and because benthic fish, being generally bigger, will be energetically richer than smaller pelagic fish [54].

Overlapping dive depths at night between fur seals and sea lions occurred in the first 40 m of the water column, suggesting that both species could be pursuing vertically migrating prey. Most of the sea lion night dives occurred within the range of 0–40 m. Fur seal night dives exhibited a bimodal distribution with a great proportion of dives within the 0–30 m range (overlapping with sea lions) and a second portion at 40–80 m depth, where almost no sea lion night dives occur. Fur seals diving behavior is influenced by the lunar cycle, increasing in depth according to lunar light intensity [55]. Although overall sea lions dive deeper (day and night) than fur seals, most of their night dives were shallower and occurred within the foraging depth range of fur seals (0–30 m). While the depth range between 40–80 m depth at night is almost exclusively exploited by fur seals, a small proportion of sea lion intermediate and deep strategy dives occurred at deeper depths not used by fur seals.

#### Time of night dives

Trillmich [42] stated that niche separation between the sympatric Galapagos fur seal and sea lion was more extensive than different habitat choice on land. While fur seals fed mostly at night and at shallow depths, sea lions did most of their feeding during the day. Consistent with Jeglinski et al. [41], we found that Galapagos sea lions also dive at night, potentially overlapping with the foraging niche of fur seals. Here, we extended the scope of previous studies by investigating niche overlap between differing foraging groups within sea lions compared to fur seals:

Sea lions exhibited the greatest percentage of their night dives around 5:00 and 18:00 (22% of total night dives) just before sunrise and after sunset; interestingly these hours were when fur seals dove the least. This is expected, as fur seals not being physiologically capable of diving to greater depths, they wait until the deep scattering layer moves closer to the surface. Fur seals exhibited the greatest percentage of night dives between 19:00–22:00 (42%). Nonetheless, there is some overlap between fur seals and sea lions, as a percentage of sea lion dives (18.5%) also occurred at 19:00 and 21:00.

Sea lion shallow divers and fur seal dives overlapped at 21:00 and 22:00, between 0–30 m depths. Sea lion intermediate divers and fur seal dives overlapped at 19:00 and 21:00. These dives occurred within 0–40 m. Correspondingly, sea lion deep divers and fur seal dives overlapped at 19:00, 20:00 and 21:00. These dives also occurred within 0–40 m depth. This sea lion group exhibited the greatest percentage of night dives (80.3%) compared to the other sea lion groups. Therefore, contrary to what we hypothesized, the deep divers group, albeit of presenting the deepest dives from all sea lion diving strategies, they also performed a great percentage of shallower dives within fur seals diving range. This sea lion strategy exhibited the greatest percentage of night dives and overlap in depth and night time with fur seals. It is possible that fur seals and sea lion deep divers are hunting for similar prey such as myctophids and cephalopods at these shallower depths. Myctophids and cephalopods are main prey items in the diet of Galapagos fur seals [23], [29] and although they have not been identified in the diet of Galapagos sea lions at their western distribution (where they coexist with fur seals), they are part of their diet in their central, southern and eastern distribution [56], [57].

At 21:00 all three groups of sea lions and fur seals are diving within the same depth range (0–30 m), and at 19:00 their mean dive depths within this range are closest. However, fur seals are exploring depths beyond 30 m, rarely explored by sea lions. Therefore, potential foraging niche overlap between fur seals and all three sea lion groups is occurring between 0–30 m depths at 19:00 and 21:00.

Although mean dive depths of all sea lion diving groups and fur seals are closest at 19:00, dive depth maximum efficiencies differed between species. Therefore, these coinciding dive depths in time are outside the depth range of their respective maximum efficiency.

Additionally, the fur seals diving behavior is influenced by the lunar cycle, increasing in depth according to lunar light intensity [55]. Sea lions are not known to be influenced by the lunar cycle. We did not consider the lunar cycle in our analysis. Furthermore, our diving data is not continuous as it comes from two separate seasons and the deployment time of each individual within each season was not long enough to follow a complete lunar cycle. Nevertheless, because fur seals exhibited a great portion of their night dives at deeper depths than sea lions, this depth range from 40–80 m (that is almost exclusively being frequented by fur seals) might disappear or become shallower if lunar cycle is considered. Therefore foraging niche overlap between sea lions and fur seals might be accentuated around and during the new moon phase.

### Spatial niche overlap

Overall, there is a spatial niche separation between species as fur seals foraging range is significantly larger than that of sea lions, partly explained by differences in their provisioning strategies as fur seals foraging trip durations are longer than those of sea lions (Villegas-Amtmann, unpubl.) [35]. However, fur seal area of highest diving density (north of the rookery – Cabo Douglas) is small and most of it is located within the sea lion area of highest diving density. Fur seal mean dive depth within this area is 21.5±20.0 m compared to 35.8±20.5 m outside the highest diving density area. Although mean dive depth inside the overlapping area is shallower than outside this area, it is still noticeably deep to be considered foraging dives. This demonstrates that the coastal area just north of Cabo Douglas is a foraging “hot spot” for both species where competition might occur.

Foraging range overlap was greater between fur seals and sea lion deep divers, coinciding with the greatest overlap in diving depth and dive hours. Differing with what we hypothesized, the sea lion deep diving strategy exhibited the greatest temporal and spatial niche overlap with fur seals.

By studying ecological interactions with an individual-based approach, we were able to detect foraging niche overlap on a finer scale that was previously overlooked. Individual specialization should be incorporated into models of food webs, competition, and predator-prey and host-parasite interactions [58].

### Future implications

Niche breadth is increased with increased environmental uncertainty and with decreased productivity [5]. The year when our study was carried out (2009) was considered a normal year with respect to El Niño oceanographic conditions, and took place after a moderate La Niña year. We found a small overlap in fur seals and sea lions diving niche as a result of temporal and spatial segregation, but foraging at different times and locations does not necessarily reduce foraging niche overlap unless these species are consuming different prey. Wolf et al. [59], Paez-Rosas et al.[23] and Jeglinski et al. [41] found trophic segregation between sea lions and fur seals at Fernandina Island based on their C and N isotopic signatures and potential trophic overlap during a moderate El Niño year. Therefore, the potential for foraging niche overlap between fur seals and sea lions is possibly greater during warmer periods when prey availability is lower, consistent with previous findings [29], [30]. Therefore, if oceans continue on a warming trend, the continuation of conservation programs for these species becomes crucial.

An additional possible response to climate change could be altered body size. Body size directly affects energy and water requirements for thermoregulation [60], energy, mass acquisition and utilization rates [61] and life-history characteristics [62]. Body-size declines are the universal response to climate change suggested by some authors [63]. Fur seals from Fernandina Island, significantly smaller than sympatric sea lions, exhibited lower foraging effort expressed as lower degree of intra-specific competition compared to sea lions. In a warming climate scenario, it is possible that fur seals have a survival advantage over sea lions either by thermoregulatory effects, given that fur seals are smaller and have a greater surface area to volume ratio from which they can lose heat or by a lower overall energy requirement.

Although when sympatric, fur seals are more successful; it is possible that its low plasticity in foraging behavior, shown by their lower individual variability, has contributed to their overall smaller population size. Compared to the highly plastic sea lions, fur seals are more impacted by variations in prey abundance, such as during El Niño events [30], [64], possibly due to a reduced diving capability added to their lower plasticity.

Furthermore, it is also possible that resource availability and preferred prey type (during normal years) for fur seals, such as cephalopods, small schooling fish, myctophids and bathylagids, [23], [29], [52] have remained more constant and stable over time, hence their unchanged diving behavior throughout the years. In contrast, sea lions foraging behavior has shown to be highly variable and plastic and sardines, their main prey type on the western populations [23], [29] (where they are sympatric with fur seals), are known to fluctuate widely in abundance over inter-annual to multi-decadal time scales [65], [66].
